# Danger of Abdominal Caesarian Section in Eclampsia

**Published:** 1913-09

**Authors:** 


DANGER OF ABDOMINAL CAESARIAN SECTION IN ECLAMPSIA, Dr. John T. Villiams, *Boston M. and S. Jour.*, March 27, 1913.

Abdominal Caesarean section was first performed in eclampsia by van den Akker in 1875 and by Halbertsma in 1889. Excluding operations performed post mortem and on the dying, I have been able to collect in all 85 cases from the literature. It is fair to state that in 21 of these there existed a mechanical indication as well as for Caesarean section, but as this in no way influenced the actual result of the operation, such cases are equally as available for our consideration as those in which eclampsia alone furnished the indication for this method of delivery.

It will be oberved that these were all cases of well developed eclampsia, therefore deductions drawn from them do not of necessity apply equally to threatened eclampsia.

The following table presents a brief resume of the reported cases. The omission of blood pressure observation is not an oversight, but it is because no mention was made of it in any reported case.

Among the 85 cases there were 41 maternal deaths, a mortality of 48.2%. This mortality is distributed as follows: From sepsis, 7 deaths; from hemorrhage from broad ligament after Porro operation, 1; from rectal hemorrhage, 1; from tuberculosis, 1; from exhaustion, 1; from pneumonia, 1; from eclampsia, 20. In 9 cases the immediate cause of death was not stated, but presumably was eclampsia.

Now let us compare these figures with the results by a few leading operators with the two other methods of rapid delivery in eclampsia.

### Manual or Instrumental Dilation.

| | | |
|---|---|---|
| Zweifel .................. | 80 cases | 15% mortality |
| Glockner ................ | 143 cases | 15.49% mortality |
| Ferri ..................... | 82 cases | 7% mortality |
| Newell ................... | 79 cases | 26.5% mortality |

### Vaginal Caesarean Section.

| | | |
|---|---|---|
| Dührssen ................ | 112 cases | 15% mortality |
| Beckmann ............... | 43 cases | 18% mortality |
| Veit ...................... | 13 cases | 3% mortality |
| Fry ....................... | 13 cases | 6.66% mortality |

The high mortality from abdominal Caesarean section results partly from peritonitis and other forms of sepsis, but mainly from the added strain thrown upon the liver and kidneys.

The fetal results demand some mention. In examining the literature it has been extremely difficult to find out the cause of fetal deaths or even to separate still-births from deaths occurring soon after birth. Friedemann gives as the general fetal mortality in 1,601 cases of eclampsia 75.4%, and Goedecks 48% in 330 cases.

As would be expected, the fetal results are better after Caesarean action (42.2% mortality) ; but so many factors—prematurity, the effect of convulsions and the toxines of eclampsia on the child—influence unfavorably the outcome that the fetal results must be poor under any treatment.

### Conclusions.

Abdominal Caesarean section in eclampsia has a maternal mortality of 48.2%.

Vaginal Caesarean has a mortality ranging from 3% to 18% ; and dilatation of the cervix from 7% to 26%.

Therefore, abdominal Caesarean section in eclampsia should be restricted to those cases in which there is a pelvic contraction sufficient in itself to demand it, and possibly also early cases of threatened eclampsia at or near term, where the shock of a vaginal delivery seems to offer much greater danger than the added strain imposed upon the excretory organs by Caesarean section.

